# Not “Much Room” in the Heart: A Rare Case of a Massive Intracardiac *Candida* Mass

**DOI:** 10.1155/2021/9216825

**Published:** 2021-07-23

**Authors:** Ali Haider Jafry, Sardar Hassan Ijaz, Murtaza Mazhar, Areeba Shahnawaz, Ali Yousif

**Affiliations:** ^1^800 Stanton L. Young Blvd, AAT 6300, Department of Medicine, University of Oklahoma Health Sciences Center, Oklahoma City 73105, OK, USA; ^2^Department of Cardiovascular Disease, Lahey Medical Center, 41 Mall Road, Burlington 01805, MA, USA; ^3^Department of Cardiovascular Disease, University of Texas Medical Branch at Galveston, 301 University Boulevard, Galveston 77555, TX, USA; ^4^Faisalabad Medical University, Sargodha Road, Faisalabad 38000, Punjab, Pakistan; ^5^Baylor Scott and White the Heart Hospital Arrhythmia Management, 1100 Allied Dr, Plano 75093, TX, USA

## Abstract

**Introduction:**

Coupled with the increasing use of indwelling vascular catheters and prosthetic cardiac valves is an uptrend in sepsis secondary to fungemia. An insidious onset often shrouds the initial diagnosis, contributing to poor outcomes. *Candida* infective endocarditis (CIE) is a feared complication of candidemia, associated with high mortality rates. It requires prolonged hospital stays for medical and, often, surgical management. We report a case of a massive intracardiac *Candida* mass in an adult with native valve CIE.

**Case:**

A 51-year-old male on chronic total parenteral nutrition (TPN) because of bowel resection presented with fevers, night sweats, and unintentional weight loss. He was febrile and tachycardiac on admission, with a benign physical examination. Laboratory workup showed elevated inflammatory markers and an acute kidney injury. Extended blood cultures showed growth of *Candida glabrata (C. glabrata)* and *Candida dubliniensis (C. dubliniensis)*. Transthoracic (TTE) and transesophageal echocardiography revealed a large mobile right atrial mass (4 cm × 6 cm × 2.5 cm), extending to the right ventricular outflow tract. Since he was a poor surgical candidate, management with micafungin was initiated and continued for 8 weeks. He responded well to the regimen with resolution of the fungal mass on follow-up TTE 3 months later. In anticipation of the future need for TPN, he continues on lifelong suppressive oral fluconazole.

**Conclusion:**

CIE may be an insidious complication of indwelling central venous catheters, necessitating a high index of suspicion. Conservative management, with antifungal therapy, can yield favorable outcomes in poor surgical candidates.

## 1. Introduction

*Candida* infective endocarditis (CIE) is a dreaded complication of candidemia and is associated with exceptionally high mortality rates, ranging from 30–80% [1, 2]. A rising incidence of fungemia places patients in an ever-increasing peril for contracting CIE, with *Candida* species causing over half of all fungal endocarditis cases [1, 3]. Indwelling vascular catheters, intravenous drug use, valvular heart disease/surgery, antibiotic use, and immunosuppressed states are potent risk factors [1, 2]. CIE with intracardiac *Candida* masses has been reported in the neonatal population but remains a rare occurrence in adults with native valves [4]. There is a lack of robust data for treatment modalities in individuals with CIE who are poor surgical candidates. We report a case of a large intracardiac *Candida* mass in a patient with native valves as a complication of total parenteral nutrition (TPN), with a favorable outcome after conservative medical management.

## 2. Case Report

**Figure 1:**
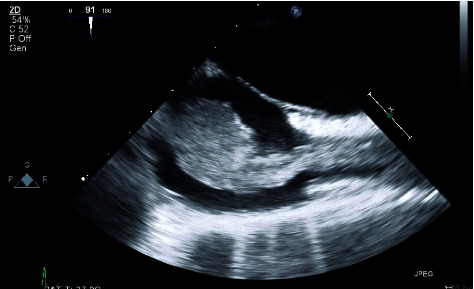
Transesophageal Echocardiogram (TEE) midesophageal bicaval view: large lobulated mobile mass extending from the superior vena cava (SVC) into the right atrium.

**Figure 2:**
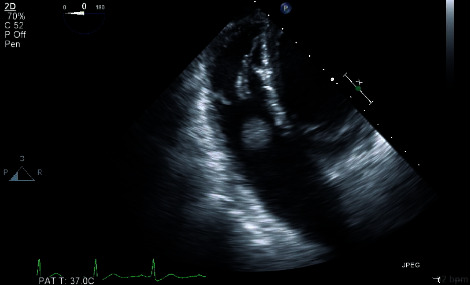
TEE-modified deep transgastric view: tip of the mass is seen at the body of the right ventricle below the tricuspid valve.

**Figure 3:**
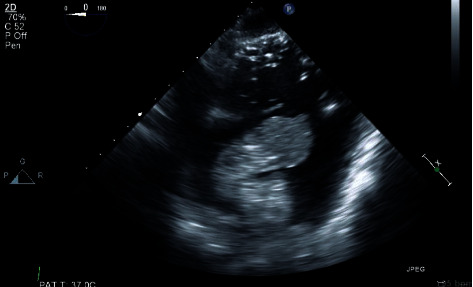
TEE-modified transgastric right ventricle inflow view: large hyperechoic lobulated mass across the tricuspid valve and occupying the body of the right ventricle.

**Figure 4:**
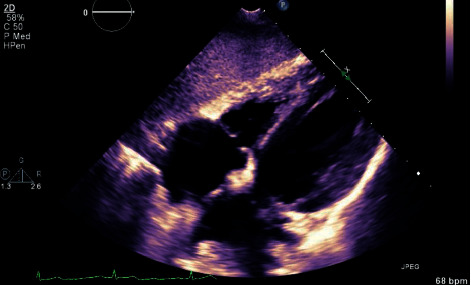
Transthoracic echocardiogram (TTE) after antifungal treatment with resolution of the large fungal mass.

A 51-year-old male on chronic TPN for short bowel syndrome after a colectomy was admitted for sepsis and acute kidney injury. He reported a month-long history of generalized fatigue, intermittent low-grade fevers, chills, night sweats, and unintentional weight loss. On presentation, he was febrile (101.6°F), tachycardiac (HR 106 bpm), but hemodynamically stable (BP: 130/86 mm Hg) and saturating well (96%) on room air. Physical examination revealed no erythema or tenderness at the site of his peripherally inserted central catheter (PICC), through which he had been receiving TPN. No significant cardiac murmurs or sequelae of infective endocarditis were observed, and the remainder of physical examination was benign as well. Admission laboratory workup revealed a white cell count of 12,000/mm^3^, elevated ESR: 99 mm/hr, CRP: 139 mg/L, and creatinine: 3.56 mg/dL (baseline: 0.9 mg/dL). Urinalysis and chest X-ray were unremarkable for infection, and standard blood cultures displayed no growth of microorganisms. Electrocardiography (EKG) showed normal sinus rhythm without any conduction system abnormalities. Broad-spectrum antibacterial coverage with vancomycin and piperacillin-tazobactam was initiated, while micafungin was added with suspicion of fungemia given the history of TPN. Extended blood cultures from admission showed growth of *Candida glabrata (C. glabrata)* and *Candida dubliniensis (C. dubliniensis)*; both species were sensitive to amphotericin-B (minimum inhibitory concentration: 0.2 mcg/ml) and micafungin (minimum inhibitory concentration: 0.12 mcg/ml). The PICC was subsequently removed; cultures from the catheter tip also grew *C. glabrata* and *C. dubliniensis*. A transthoracic echocardiogram (TTE) showed an echogenic large mobile right atrial mass (4 cm × 6 cm × 2.5 cm), extending from the superior vena cava into the right atrium and prolapsing into the right ventricle and right ventricular outflow tract. There was no evidence of outflow tract obstruction. These findings were concerning for an intracardiac fungal mass versus a large thrombus. Transesophageal echocardiogram (TEE) revealed similar lesions, with an additional aortic valve vegetation (1 cm × 0.5 cm) (Figures 1–3). EKGs remained unremarkable, and ophthalmologic exam was not concerning for endophthalmitis. A computed tomography (CT) scan of the chest showed diffuse nodular opacities in the lungs bilaterally, concerning for septic emboli. Cardiothoracic surgery was consulted for surgical intervention for the intracardiac mass. However, due to the patient's significant comorbidities and extensive size of the lesion, he was deemed a poor surgical candidate. He was, thus, treated conservatively with micafungin for 8 weeks. Following acute antifungal therapy and given the need for ongoing chronic TPN, he was started on lifelong suppressive therapy with fluconazole after sepsis resolved. Three months later, a repeat TTE showed complete resolution of the intracardiac mass without evidence of persistent vegetations, thus confirming the fungal origin of the mass (Figure 4).

## 3. Discussion

The increasing use of indwelling vascular catheters, intracardiac devices, and prosthetic cardiac valves has contributed to an upsurge of candidemia and CIE, countered to an extent by the approval of echinocandins in 2003, which are now recommended as first-line agents [3, 5]. Most cases of CIE are nosocomial in origin (67%); total parenteral nutrition (TPN) is a significant etiology for right-heart CIE [2]. Presenting complaints may be subtle and commonly consist of constitutional symptoms such as fevers, chills, night sweats, generalized fatigue, and weight loss. This often leads to delayed or mistaken diagnosis (up to 82% of patients), further complicating patient care, with prior studies reporting a 32-day gap between symptom onset and hospitalization [6].

Cardiac involvement may result in worsening or new-onset congestive heart failure due to outflow tract obstruction or valvular compromise, depending on the size or site of the mass. Other complications involve paravalvular leaks, intracardiac abscesses, and septic embolization; the latter may result in endophthalmitis, cerebrovascular accidents, osteomyelitis, etc. Fungal vegetations tend to be larger in size and carry an increased risk of embolization compared to bacterial vegetations [7]. Intracardiac fungal masses are a rare complication of CIE. Mainly reported in preterm neonates with candidemia, cases in adults are rare, limited to patients with prosthetic valves or intracardiac devices [4]. In our case, the unique aspect was the occurrence of a large valvular mass in an adult without outflow obstruction, which is rarely seen in adults with *Candida* CIE.

Available data for the treatment of fungal endocarditis, particularly involving intracardiac fungal masses, is based primarily on case reports, case series, and observational studies. The recommended practice per the Infectious Diseases Society of America involves adjunctive surgery wherever possible to achieve source control along with an appropriate antifungal regimen [5]. Recent prospective studies, however, do not demonstrate mortality benefit with a combined medical/surgical option compared to medical therapy alone [2, 8]. This bears particular importance for patients who may be poor surgical candidates. Corroborating this, despite the extent of the intracardiac *Candida* mass in our case and inability to undergo surgical management, antifungal medications yielded a favorable outcome.

Initial treatment regimens consist of parenteral amphotericin-B or an echinocandin. Animal models and prospective studies have shown both regimens to be equally effective [2, 9]. Patients often require a prolonged suppressive regimen comprising an azole, commonly fluconazole, based on sensitivities. If surgery is not possible or underlying risk factors are expected to persist, lifelong suppressive therapy with fluconazole is employed to prevent the relapse of infection and improve patient survival [10, 11].

## 4. Conclusions

The diagnosis of CIE requires a high degree of clinical suspicion owing to its insidious onset and high possibility of initial misdiagnosis. In poor surgical candidates, a prolonged course of antifungals followed by chronic suppressive therapy with fluconazole can yield favorable outcomes, even without surgical management. Clinicians must remain vigilant against drug resistance as a possible drawback of chronic antifungal therapy.

## Data Availability

The data that support the findings of this study are available from the corresponding author upon reasonable request.
